# Local Injection of Submicron Particle Docetaxel is Associated with Tumor Eradication, Reduced Systemic Toxicity and an Immunologic Response in Uro-Oncologic Xenografts

**DOI:** 10.3390/cancers11040577

**Published:** 2019-04-24

**Authors:** Holly A. Maulhardt, Lauren Hylle, Michael V. Frost, Ashley Tornio, Sara Dafoe, Leanne Drummond, David I. Quinn, Ashish M. Kamat, Gere S. diZerega

**Affiliations:** 1 US Biotest, Inc., 231 Bonetti Drive, Suite 240, San Luis Obispo, CA 93401, USA; holly.maulhardt@usbiotest.com (H.A.M.); lauren.peterson@usbiotest.com (L.H.); ashley.tornio@usbiotest.com (A.T.); sara.dafoe@usbiotest.com (S.D.); leanne.drummond@usbiotest.com (L.D.); 2 Western Diagnostic Services Laboratory, 1414 East Main Street, Suite 102, Santa Maria, CA 93454, USA; mfrost@wdslaboratory.com; 3 Department of Medicine, The University of Southern California Norris Cancer Center, 1441 Eastlake Avenue, Los Angeles, CA 90033, USA; diquinn@med.usc.edu; 4 Department of Urology, University of Texas MD Anderson Cancer Center, 1515 Pressler, Unit 1373, Houston, TX 77030, USA; akamat@mdanderson.org; 5 NanOlogy, LLC., 3909 Hulen Street, Fort Worth, TX 76107, USA

**Keywords:** docetaxel, NanoDoce^®^, intratumoral, nanoparticle, genitourinary oncology, cancer, bladder, renal, prostate

## Abstract

Intratumoral (IT) administration of submicron particle docetaxel (NanoDoce^®^, NanOlogy LLC, Fort Worth, TX, USA) and its efficacy against genitourinary-oncologic xenografts in rats and mice, xenograft-site docetaxel concentrations and immune-cell infiltration were studied. IT-NanoDoce^®^, IV-docetaxel and IT-vehicle were administered to clear cell renal carcinoma (786-O: rats), transitional cell bladder carcinoma (UM-UC-3: mice) and prostate carcinoma (PC-3: mice). Treatments were given every 7 days with 1, 2, or 3 doses administered. Animals were followed for tumor growth and clinical signs. At necropsy, 786-O and UM-UC-3 tumor-site tissues were evaluated by H&E and IHC and analyzed by LC-MS/MS for docetaxel concentration. Two and 3 cycles of IT-NanoDoce^®^ significantly reduced UM-UC-3 tumor volume (*p* < 0.01) and eliminated most UM-UC-3 and 786-O tumors. In both models, NanoDoce^®^ treatment was associated with (peri)tumor-infiltrating immune cells. Lymphoid structures were observed in IT-NanoDoce^®^-treated UM-UC-3 animals adjacent to tumor sites. IT-vehicle and IV-docetaxel exhibited limited immune-cell infiltration. In both studies, high levels of docetaxel were detected in NanoDoce^®^-treated animals up to 50 days post-treatment. In the PC-3 study, IT-NanoDoce^®^ and IV-docetaxel resulted in similar tumor reduction. NanoDoce^®^ significantly reduced tumor volume compared to IT-vehicle in all xenografts (*p* < 0.0001). We hypothesize that local, persistent, therapeutic levels of docetaxel from IT-NanoDoce^®^ reduces tumor burden while increasing immune-cell infiltration. IT NanoDoce^®^ treatment of prostate, renal and bladder cancer may result in enhanced tumoricidal effects.

## 1. Introduction

Local treatment of solid tumors has the potential to overcome the limitations of conventional intravenous (IV) chemotherapy. These limitations include rapid disposition throughout the patient after administration, resulting in shorter chemotherapeutic tumor dwell time and systemic toxicities. It has long been hypothesized that successful local tumor treatment would increase local drug concentrations over multiple cell-division cycles and reduce systemic toxicity, thus providing tumoricidal benefits without compromising the patient’s well-being [1].

Nanoparticle-based drug delivery systems are among those being developed for treatment of cancer. However, success of these systems may be limited by their systemic administration and abbreviated tumor residence due, in part, to the clearance of nanoparticles by phagocytes [2]. Nanoparticle taxanes (NanoDoce^®^ and NanoPac^®^; CritiTech, Inc., Lawrence, KS, USA) were developed to increase intratumoral drug residence time through local delivery of submicron particles. NanoDoce^®^ is produced using submicron particle production technology that sonicates dissolved docetaxel drug substance into uniform droplets containing 2–3 billion docetaxel molecules. The solvent is stripped from the solution using supercritical fluid carbon dioxide which precipitates the pure particles of docetaxel (~900 nm). Conventional IV docetaxel is standard-of-care treatment for metastatic bladder and prostate cancer and NanoDoce^®^ is currently being studied in a clinical trial in the local treatment of genitourinary neoplasms (NCT03636256). Similar submicron particles of paclitaxel (NanoPac^®^; NCT00666991 [3], NCT03029585, NCT03077659, NCT03077685, NCT03188991) are under clinical evaluation for other cancers. Development of inhaled NanoPac^®^ for treatment of non-small cell lung cancer is ongoing [4].

In response to IV chemotherapy, cancer cells release a spectrum of cell death-associated or immunologically activating molecules [5,6,7,8]. Tumor cell necrosis may provide immunological stimulation that is both proinflammatory and immunogenic [9,10,11,12,13]. Docetaxel has been shown to favorably mediate the anti-cancer response of macrophages, CD8^+^ T cells, B cells and natural killer cells (NKs). When peripheral blood mononuclear cells and macrophages from healthy donors were incubated with 1–10 µM docetaxel they subsequently displayed an activated phenotype characterized by increased expression of HLA-DR and CD86 [14]. Preclinical studies of docetaxel show increased cytotoxic activity of T cells and infiltration of immune cells into the tumor microenvironment [15]. Docetaxel has similar effects on B cells; its effects as a vaccine adjuvant induce IgG secretion and increase B cell proliferation and tumor infiltration [16]. Additionally, docetaxel has been reported to inhibit myeloid-derived suppressor cells in tumor bearing mice [17]. In advanced breast cancer patients, 6 cycles of IV docetaxel treatment resulted in a nearly 250% increase in NK cytotoxicity [18].

Findings from our preclinical evaluations in genitourinary-oncologic xenografts in rats and mice demonstrated substantial reduction of tumor size after IT injection of NanoDoce^®^, which was superior (in renal and bladder tumors) or similar (in prostate tumors) to results from IV docetaxel administration. Further, in contrast to IV docetaxel, reduction in tumor size was maintained following IT NanoDoce^®^. Systemic toxicity, as measured by body weight and clinical signs, was generally reduced in IT NanoDoce^®^ compared to IV docetaxel-treated groups. Docetaxel was detectable in all evaluated tumor tissue up to 50 days post-IT NanoDoce^®^ administration. Histological analysis confirmed that NanoDoce^®^ was associated with tumor regression. Immunohistochemistry (IHC) demonstrated an association between IT NanoDoce^®^ and the presence of CD68^+^ and CD11b^+^ stromal-infiltrating cells as well as lymphoid structures (LS).

## 2. Results

### 2.1. Tumor Volume

**Figure 1:**
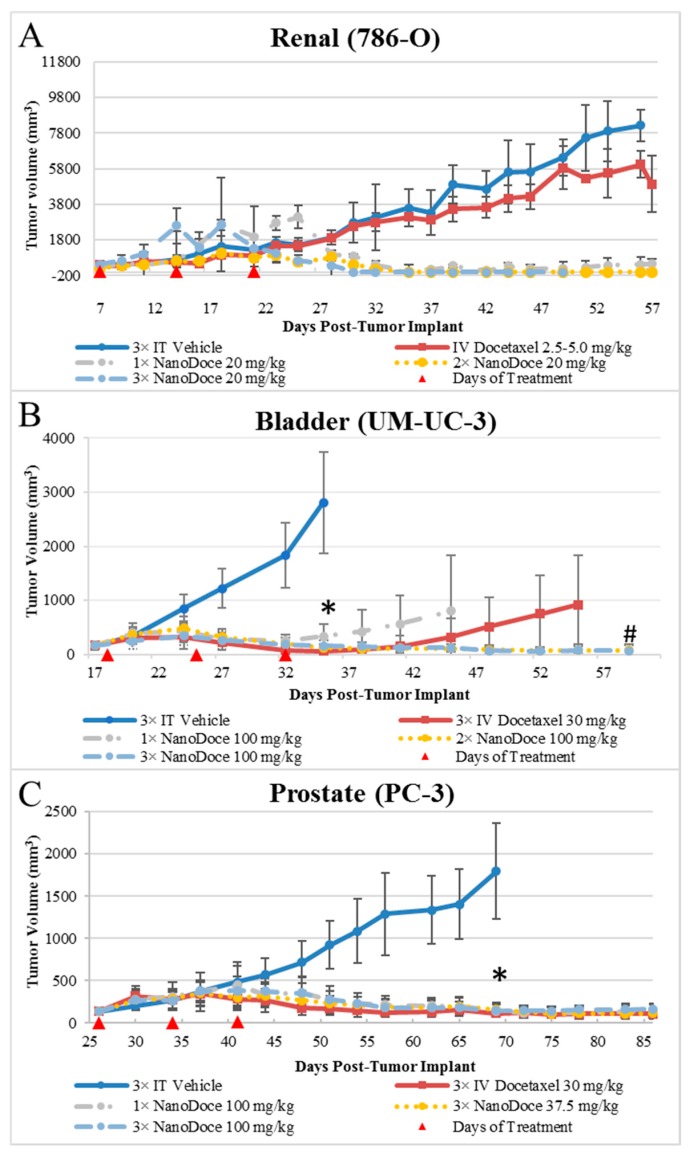
Group mean TVs from treatment initiation to study end. Data plotted through point when ≥50% of animals in group survived. Error bars = ±1 Std. dev. (**A**) 786-O xenografts; *n* = 3/group; treatment was initiated 7 days after tumor implant when group mean TVs ranged from 336–427 mm^3^. (**B**) UM-UC-3 xenografts; *n* = 9 or 10/group; treatment was initiated 18 days after tumor implant when group mean TVs ranged from 161–164 mm^3^. * *p* < 0.0001 vs. 3× IT Vehicle; # *p* < 0.01 for 3× IV Docetaxel 30 mg/kg vs. 2× and 3× NanoDoce^®^ 100 mg/kg groups. (**C**) PC-3 xenografts; *n* = 10/group; treatment was initiated 26 days after tumor implant when group mean TVs ranged from 136–141 mm^3^. * *p* < 0.0001 vs. 3× IT Vehicle. Statistical significance was determined using one-way ANOVA with Dunnett’s post-test analysis. In all studies, red triangles designate days of treatment.

Tumor growth data for all xenografts are presented in Figure 1. All treatments of 786-O xenografts resulted in tumor inhibition relative to untreated and vehicle control. IT NanoDoce^®^ produced a nearly complete and sustained reduction in tumor volume (TV); in some cases where three cycles were administered, complete eradication of tumor was observed with a scab remaining at the treatment site. In contrast, IV docetaxel failed to reduce tumor size, with resulting tumor volumes similar to vehicle controls. Due to the small number of animals per group (*n* ≤ 3), statistical comparisons were not possible. Similarly, all treatments of UM-UC-3 xenografts resulted in significant tumor reduction compared to vehicle control (Figure 1; *p* < 0.0001; Day 35). Both 2× and 3× IT NanoDoce^®^ (100 mg/kg) groups exhibited tumor inhibition throughout the study. At study end (Day 61), mean tumor volume in these groups was significantly less than IV docetaxel and 1× IT NanoDoce^®^ (*p* < 0.01) and IV docetaxel and 1× IT NanoDoce^®^ groups had an increase in TV approximately 2 weeks after treatment cessation. One animal from both the IV docetaxel and 1× IT NanoDoce^®^ groups were euthanized early due to tumor growth. In the UM-UC-3 study, one animal from both the IV docetaxel and 1× IT NanoDoce^®^ groups and two animals treated with 2× IT NanoDoce^®^ exhibited metastases.

In both the 786-O and UM-UC-3 models, treated animals were maintained on study beyond the days at which vehicle controls reached their defined endpoint to allow for assessment of tumor reduction durability and for analysis of docetaxel in tumor tissues at times far removed from treatment administration. All treatments of PC-3 xenografts resulted in significant tumor reduction compared to vehicle control (Figure 1; *p* < 0.0001). Partial tumor regression was observed in at least one animal in groups treated with IV docetaxel or IT NanoDoce^®^ and the mean TVs between these groups were not significantly different.

### 2.2. Toxicity

Ulceration at the tumor site was noted in animals from both the 786-O and UM-UC-3 studies. In the 786-O study scabs and areas of dry, rigid tissue covering 10–50% of the tumor surface were observed. Two animals from this study were euthanized early due to the extent of ulceration. In the UM-UC-3 study, increased IT NanoDoce^®^ administrations resulted in greater incidence of ulceration at the site of injection. Tumor site tissue damage occurred in 89% of animals administered 1× and 2× IT NanoDoce^®^ (8/9 per group) and 100% (9/9) of animals that received 3× IT NanoDoce^®^. Tumor site tissue damage occurred in 30% (3/10) of animals in the IT vehicle control group. There were no reports of tumor ulceration in the PC-3 study.

In the 786-O study, immediately following the second administration of IV docetaxel, (5 mg/kg), one animal stopped breathing and expired while under anesthesia. The next animal administered IV docetaxel had a temporary loss of respiration and slower than normal recovery from anesthesia. Due to these signs of toxicity, the third animal in the cohort was not administered a second cycle of IV docetaxel and, at the third cycle, the dose was reduced to 2.5 mg/kg for the two remaining animals. A single animal in the 786-O xenograft study that received 3× IT NanoDoce^®^ exhibited hindlimb weakness and limited mobility after treatment cessation. The animal was treated per veterinary recommendations and the weakness stabilized sufficiently to remain in the study.

**Figure 2:**
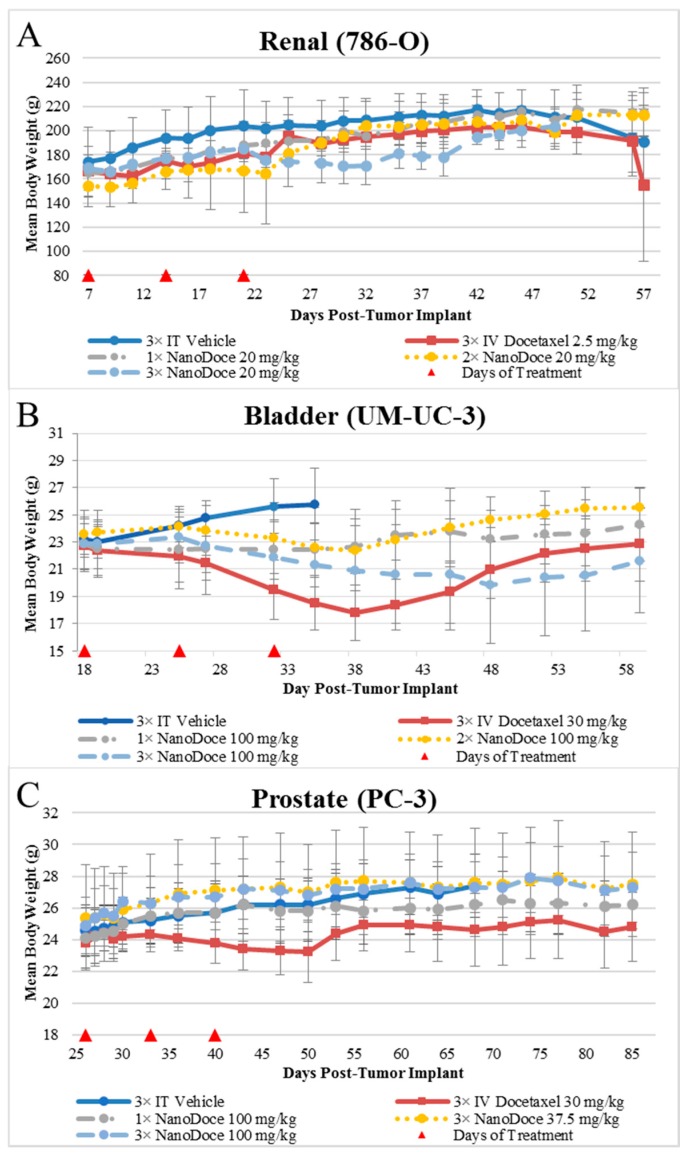
Mean body weights from treatment initiation through study end. Data plotted through point when ≥50% of animals in group survived. Error bars = ±1 Std. Dev. (**A**) Female rats implanted with 786-O tumors; *n* = 3/group. (**B**) Female mice implanted with UM-UC-3 tumors; *n* = 9 or 10/group. (**C**) Female mice implanted with PC-3 tumors; *n* = 10/group.

Mean body weight values are presented in Figure 2. Body weights among all rats with 786-O xenografts were similar, with two exceptions. The 3× IT NanoDoce^®^ group exhibited a lower mean body weight 4 to 18 days after treatment cessation. This was largely affected by a single animal that exhibited weight loss >20% and was euthanized 18 days after final treatment. An animal treated with 2× IT NanoDoce^®^ was euthanized 16 days after treatment initiation due to weight loss >20%. In the UM-UC-3 study, IT vehicle treated animals were available for body weight evaluation through Day 35 after which >50% of the animals in the cohort were euthanized for reaching study endpoint (TV > 3000 mm^3^). Animals implanted with UM-UC-3 tumors and treated with IV docetaxel or 3× IT NanoDoce^®^ exhibited mean weight loss which reversed after cessation of treatment (Figure 2). In the PC-3 study the IV docetaxel group also showed mean weight loss (Figure 2).

### 2.3. Survival

In the 786-O study, one animal in the IV docetaxel group died following drug administration. Early euthanasia due to weight loss occurred in one animal in each the 2× and 3× IT NanoDoce^®^ groups on Days 16 and 39, respectively. Two animals in the 3× IT NanoDoce^®^ group were euthanized at Day 50 due to tumor site ulcerations. In the UM-UC-3 study vehicle control animals survived to Day 52, significantly less (*p* < 0.001; Kaplan-Meier method with Gehan-Breslow post-hoc analysis) than all other groups who survived to study end (Day 61), with the exception of one each from the IV docetaxel and 1× IT NanoDoce^®^ groups who were euthanized early due to tumor growth. In the PC-3 study, only one vehicle-treated animal and all animals treated with IV docetaxel or IT NanoDoce^®^ survived to study end (Day 86).

### 2.4. Docetaxel Concentration in Tumor Tissues

Thirty-eight days following the last administration of IV docetaxel, neither of the 786-O xenografts harvested had detectable (LOQ = 1.00 ng/g) docetaxel. In contrast, docetaxel levels measured in tumors following IT NanoDoce^®^ were high (up to 5.26 mg/g). Fifty days following 1× IT NanoDoce^®^ administration all samples had detectable docetaxel ranging from 659 ng/g to 144 μg/g. Forty-three days after 2× IT NanoDoce^®^, docetaxel levels were 2.49 and 5.26 mg/g. Tumors treated with 3× IT NanoDoce^®^ were not analyzed.

Forty-four days after IV docetaxel, one of seven UM-UC-3 tumors had detectable docetaxel (5.10 ng/g). In contrast, docetaxel levels measured in tumors following IT NanoDoce^®^ treatment were high (up to 6.70 mg/g). Forty-four days after 1× IT NanoDoce^®^, 10 of 10 tumors had docetaxel levels ranging from 154 ng/g to 2.14 mg/g. Thirty-seven days after 2× IT NanoDoce^®^, five of five tumors had docetaxel concentrations ranging from 285 ng/g to 873 µg/g. Thirty days after the 3× IT NanoDoce^®^, five of five tumors had docetaxel concentrations ranging from 373 µg/g to 6.70 mg/g.

### 2.5. Histopathology

#### 2.5.1. Renal Cell Carcinoma Xenograft

**Figure 3:**
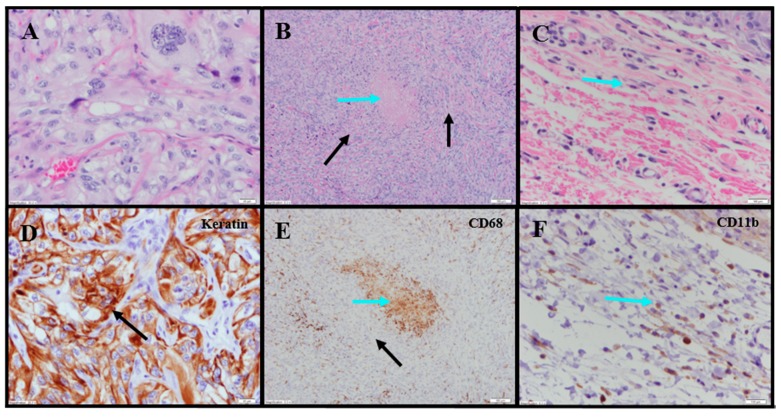
Representative photomicrographs of untreated renal tumors. (**A**) Cohesive tumor cells (TCs) 40×. (**B**) Same animal as panel (**A**): Central focus of necrosis is sharply demarcated from the surrounding viable TCs (black arrows), and is composed of amorphous material (blue arrow) 10×. (**C**) Same animal as panel (**A**): Limited, small peritumoral lymphocytes (blue arrow) 10× (cropped). (**D**) Same tumor section as panel (**A**): anti-AE1/AE3 was sensitive and specific for TCs (black arrow) 40×. (**E**) Same tumor section as panel (**B**): anti-CD68 shows limited macrophages (Mϕ) among viable TCs (black arrow) and markedly increased Mϕ in the focus of necrosis (blue arrow) 10×. (**F**) Same tumor section as panel (**C**): peritumoral CD11b+ lymphoid cells (blue arrow) 10× (cropped).

Evaluation of histopathology of tumor-site tissues from two untreated animals in the 786-O study revealed a dense nodule of invasive carcinoma (Figure 3A). Scattered discrete foci of coagulative tumor cell necrosis were present (<5% of tumor area; Figure 3B). A representative mitotic count performed on one of the animals in this group, shown in Figure 3C, had 13 mitoses per 10 high power fields (400×).

IHC staining for pan-cytokeratin was sensitive and specific (Figure 3D). There was neither overt tumor regression nor significant intratumoral lymphoid infiltrate noted in the untreated animals. Anti-CD68 showed a mild macrophage infiltrate within and around the tumor with increased density within foci of tumor necrosis, consistent with increased macrophages in areas containing necrotic cellular debris (Figure 3E). Anti-CD11b (macrophage, DC and NK cells) highlighted mild immune-cell infiltrate in the surrounding non-neoplastic stroma with no staining in the tumor (Table S1; Figure 3F). The tumor tissues from IT vehicle- or IV docetaxel-treated animals demonstrated morphologic and IHC appearances that were essentially identical to untreated controls. Maximum tumor dimensions for untreated, vehicle or IV docetaxel-treated animals ranged from 9–15 mm.

Tumor-site tissues from eight IT NanoDoce^®^-treated animals were evaluated and residual carcinoma was observed in four: two were administered 1× IT NanoDoce^®^ and their maximum cross-sectional tumor dimensions were 5 mm; the other two were administered two cycles and their maximum cross-sectional dimensions were 3 and 0.9 mm. Where present, the morphology of the tumor cells was similar to the non-NanoDoce^®^-treated groups. No residual viable carcinoma was observed in the remaining four animals administered 1× (*n* = 1), 2× (*n* = 1) or 3× (*n* = 2) cycles of IT NanoDoce^®^.

**Figure 4:**
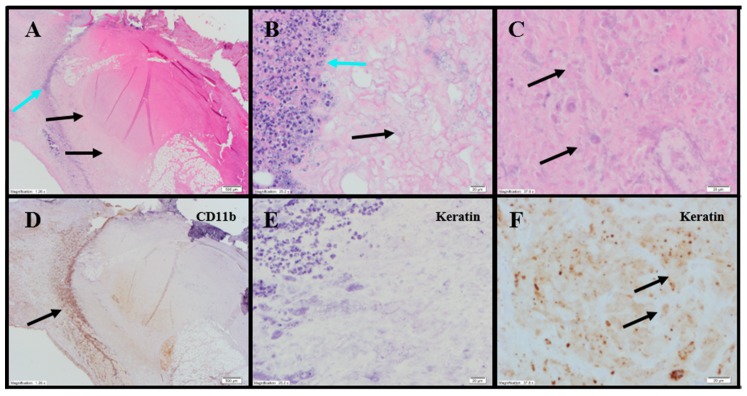
Representative photomicrographs of renal tumors administered 1× IT NanoDoce^®^. (**A**) Skin ulceration (top right). Extensive tumor cell necrosis (TCN) (black arrows) with a band of necrotic debris and admixed immune cells (ICs) (blue arrow) between the necrotic tumor and inflamed host tissue 2×. (**B**) Line of demarcation shows a dense collection of ICs, admixed debris (blue arrow) and adjacent necrotic material with no viable tumor (black arrow) 40×. (**C**) Central necrosis in (**A**): ghost outlines of necrotic tumor cells (TCs) (black arrows) 60×. (**D**) Same as panel (**A**): anti-CD11b highlights the dense IC infiltrate (black arrow) 2×. (**E**) Same band of necrotic debris as panel (**B**): complete absence of anti-AE1/AE3labelling 40×. (**F**) Similar section of tissue as panel (**C**): anti-AE1/AE3 shows degenerating keratin filaments in the necrotic ghost-cell outlines (black arrows) supporting theory that previously viable carcinoma underwent complete regression and necrosis 60×.

**Figure 5:**
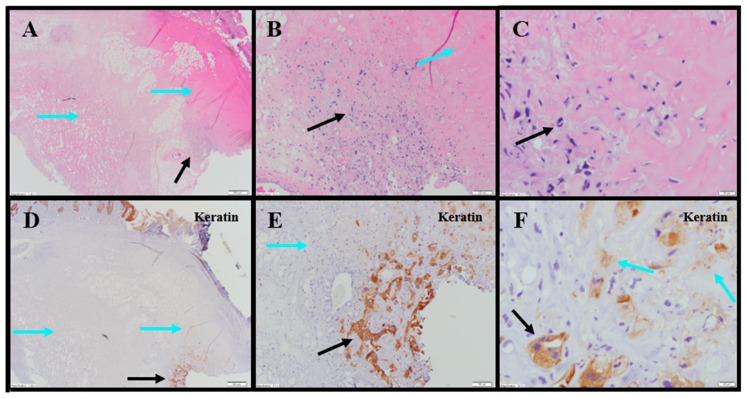
Representative photomicrographs of renal tumors administered 2× IT NanoDoce^®^. (**A**) Extensive skin ulceration (top right) above extensive necrotic material (blue arrows) and a 0.9 mm residual focus of viable carcinoma (black arrow) 2×. (**B**) Viable TCs with retained nuclei (black arrow). A progressive loss of viable TCs toward the upper right corner (blue arrow) 10×. (**C**) The leading edge of the viable tumor (black arrow) and the adjacent zone of TC death. Remnants of TCs (from bottom left (black arrow) to top right) in progressive stages of death, evidenced by loss of nuclei and discrete cytoplasmic membranes 40×. (**D**) Anti-AE1/AE3 reveals focus of residual viable carcinoma (black arrow) surrounded by an extensive unstained (necrotic) area (blue arrows). Discrete staining of normal epidermis seen at top left 2×. (**E**) Viable nucleated TCs strongly labeled with anti-AE1/AE3 (black arrow) surrounded by unstained necrotic tissue (blue arrow) 10×. (**F**) Progressive transition from viable, nucleated, keratin-positive TCs (black arrow) to TCs in varying stages of necrosis (blue arrows) 40×.

**Figure 6:**
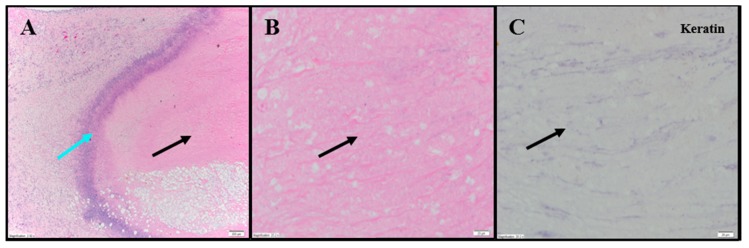
Representative photomicrographs of renal tumors administered 3× IT NanoDoce^®^. (**A**) Dense amorphous necrosis (black arrow) demarcated from surrounding fibrofatty tissue by a band of necrotic debris and admixed ICs (blue arrow) 4×. (**B**) Necrotic area from (**A**): no viable nucleated TCs (black arrow) 40×. (**C**) Same as B: absence of residual carcinoma evidenced by lack of anti-AE1/AE3 staining (black arrow) 40×.

Extensive tumor cell necrosis was observed in one animal administered 1× IT NanoDoce (Figure 4A), all animals who received two cycles (Figure 5A–F), and both animals who received three cycles (Table S2; Figure 6A–C). Anti-AE1/AE3 revealed foci of non-viable, anuclear, ghost tumor-cell outlines (Figure 4F and Figure 5F) consistent with labelling of degenerating keratin intermediate filaments in dead tumor cells.

NanoDoce^®^-treated animals exhibited no significant IT lymphoid infiltration. CD11b staining of tumor-site tissues highlighted an increased density of stromal lymphohistiocytic infiltrate with increasing cycles of treatment (Table S1; Figure 4)

#### 2.5.2. Transitional Cell Bladder Carcinoma Xenograft

**Figure 7:**
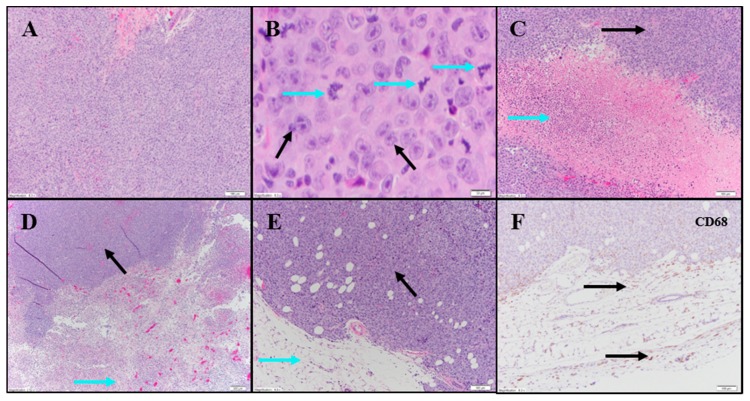
Representative photomicrographs of bladder tumors. Row 1: Untreated carcinoma. (**A**) Sheets of TCs 10×. (**B**) Same animal as in (**A**): Large TCs with prominent nucleoli (black arrows) and a marked increase in mitotic figures (blue arrows) 40×. (**C**) Same animal as in (**A**): Area of TCN (blue arrow) flanked by viable carcinoma (black arrow) 10×. Row 2: (**D**) IT vehicle: extensive necrosis in bottom half of image (blue arrow) and viable carcinoma in top left (black arrow) 4×. (**E**) IV Docetaxel: Viable carcinoma (black arrow). Surrounding fatty tissue with a sparse immune cell infiltrate (blue arrow) 10×. (**F**) Anti-CD68 shows mild macrophage infiltrate in surrounding stroma (black arrows) 10×.

Evaluation of H&E and IHC stains performed on tumor-site tissue from an untreated animal in the UM-UC-3 study exhibited extensive diffuse proliferation of invasive carcinoma that measured up to 15 mm (Figure 7A) with markedly increased mitotic activity (122 mitoses per 10 fields [10× cropped]; Figure 7B). Scattered areas of coagulative necrosis occupying 5–10% of the tumor area were noted (Table S3; Figure 7C). Necrotic foci consisted of coalesced amorphous necrotic debris containing foci of admixed degenerating tumor cells (Figure 7C).

Tumor tissues from IT vehicle or IV docetaxel-treated animals demonstrated essentially identical morphologic and IHC appearance to the untreated control tissue. Maximum cross-sectional tumor dimensions from untreated, vehicle, or IV docetaxel-treated animals ranged from 9–24 mm. Both animals administered IT vehicle exhibited geographic areas of necrosis that occupied 11–50% and 50–90% of the tumor, respectively (Table S3; Figure 7D). Where present, non-neoplastic tissue contained a mild immune-cell infiltrate. Tumors from animals treated with IV docetaxel exhibited tumor cell necrosis that occupied 11–50% and 50–90% of the tumor area (Table S3; Figure 7E,F). There was no significant lymphoid infiltrate within the untreated, vehicle or IV docetaxel-treated tumors. Anti-CD68 (Table S4; Figure 7F) highlighted a mild macrophage infiltrate within and around the tumor with increased density of staining within the foci of tumor necrosis, consistent with increased macrophage concentration in areas containing increased cellular debris (Table S4). There were no LS present in the tissues evaluated.

**Figure 8:**
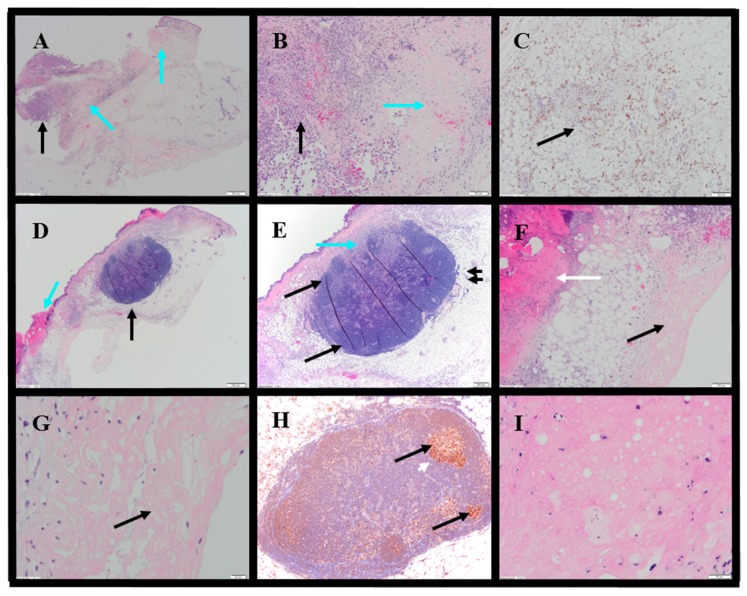
Representative photomicrographs of bladder tumors. Row 1: 1× IT NanoDoce^®^. (**A**) H&E: extensive TCN (blue arrows). Focal viable carcinoma measuring 2.5 mm (black arrow) 2×. (**B**) Extensive TCN in panel (**A**)—viable TCs (black arrow) and necrosis (blue arrow) 10×. (**C**) Anti-CD68 shows mild macrophage infiltrate (black arrow) in the surrounding fatty tissue 10×. Row 2: 2× IT NanoDoce^®^. (**D**) Skin ulceration (blue arrow) with underlying necrosis. A lymphoid structure (LS (black arrow)) 2×. (**E**) LS: discrete demarcation from the surrounding fat (double black arrows). LS organization includes a hilar region that contains sinuses (blue arrow) and lymphoid follicles (black arrows) 4×. (**F**) Same as in (**D**)—ulcerated skin at left (white arrow), necrotic tissue at right (black arrow) 10×. Row 3: (**G**) Necrotic area in panel F shows homogenous amorphous necrotic material with no viable TCs 40×. (**H**) 3× IT NanoDoce^®^: Anti-BCL6 shows LS containing lymphoid follicles composed of BCL6^+^ germinal centers (black arrows) surrounded by mantle zone B-cells that do not express BCL6 (white arrow) 10×. (**I**) 3× IT NanoDoce^®^: Area of amorphous necrotic material with no viable TCs 60×.

Histopathology evaluations were conducted on tumor-site tissues from 11 animals administered IT NanoDoce^®^ (Figure 8). Residual viable carcinoma was identified in six of these animals: three treated with 1× IT NanoDoce^®^ (maximum cross-sectional tumor dimension 2.5–19 mm) and three receiving 2× IT NanoDoce^®^ (maximum cross-sectional tumor dimension 0.1–8 mm). The remaining five animals had no residual viable carcinoma observed on the slide. Extensive tumor cell necrosis was observed in tumor-site tissues from 8/11 IT NanoDoce^®^-treated animals: one receiving 1× (Figure 8A–C), four receiving 2× (Figure 8D–G) and all three who received 3× (Table S3; Figure 8H,I).

IT NanoDoce^®^-treated tumors exhibited mild and moderate stromal immune-cell infiltrate (Table S4). Additionally, well-demarcated LS were observed in animals administered one or more cycles of IT NanoDoce^®^ (Figure 8D,E,H). A single LS was present in seven of the eight animals showing complete tumor regression or residual tumor of ≤2.5 mm, and these LS were in close proximity to the harvested tumor-site tissues. The eighth animal showing prominent regression had a 0.2 mm lymphoid collection.

## 3. Discussion

When IV chemotherapy is administered, immunosuppression often follows, counteracting immunologic effects and contributing to patient morbidity. Hence, reducing systemic exposure to chemotherapy while enhancing local tumoricidal effects remains a high priority in cancer therapy development. Our studies of locally administered submicron particle docetaxel (NanoDoce^®^) showed that multiple administrations resulted in tumor reduction in xenograft models of 786-O (human renal cell carcinoma), UM-UC-3 (human transitional cell bladder carcinoma) and PC-3 (human prostate carcinoma). In contrast, treatment with multiple cycles of IV docetaxel resulted in no (renal cell) or unsustainable (bladder) tumor volume reduction. Body weight loss, an indicator of systemic toxicity following chemotherapy, occurred in the IV docetaxel-treated group in the bladder UM-UC-3 and prostate PC-3 xenograft groups, while toxicity following IV administration required dose modification in the rat renal 786-O xenograft model. Mice administered one or two cycles of IT NanoDoce^®^ had body weight gain similar to vehicle control groups in the bladder and prostate cancer xenograft studies, while sporadic negative, non-dose dependent effects to body weight were observed in IT NanoDoce^®^-treated rats in the renal xenograft evaluations. In the bladder tumor xenografts, three cycles of NanoDoce^®^ resulted in body weight loss that recovered following cessation of treatment. A limitation of this study was that peripheral blood were not evaluated for docetaxel levels, so the systemic docetaxel exposure was not assessed. Overall, the body-weight findings and toxicities at time of dosing suggest that systemic toxicity is reduced with IT administrations of NanoDoce^®^ compared to IV docetaxel.

Systemic toxicities resulting from IV taxane therapy are well described. The generalized nature of IV chemotherapy exposes the whole body to cytotoxic drug. Hence, doses must be limited, and administration frequency minimized. In the present study, IV docetaxel-treated tumor tissues had very low (5.10 ng/g) docetaxel levels 50 days post-treatment. However, after a single IT administration of NanoDoce^®^, docetaxel was detectable in both renal and bladder tumors in concentrations ranging from 154 ng/g to 2.14 mg/g. IT NanoDoce^®^ administration resulted in intratumor docetaxel levels 30 to 500,000 times greater than IV administration. Achieving such levels at the tumor site may enhance the tumoricidal effect of docetaxel while minimizing systemic toxic events.

It appears that local administration of NanoDoce^®^ is associated with an immune response that persists at least 7 weeks after cessation of treatment. While a quantitative analysis of the histology findings was not performed, qualitative IHC analysis of tissues collected from the tumor implantation sites 30–50 days post-treatment cessation showed more stromal infiltration by immune cells in the NanoDoce^®^-treated animals compared to the IV docetaxel-treated, vehicle control and untreated animals. This infiltration was composed of CD68^+^ and CD11b^+^ cells. The lymphocytic stromal and tumor infiltration observed in tissues treated with IT NanoDoce^®^, including the presence of LS near the tumor site, suggest that locally administered NanoDoce^®^ enhances the immune response to the tumor [19]. This immune infiltration is notable for multiple reasons. First, the xenograft models utilized in these studies were deficient in T cells while macrophages and DCs were present. The rats used in the renal model lack mature B cells and have a low NK population; mice in the bladder model are also deficient in NKs but should have mature B cells. Despite the limited immune repertoire of these models, immune-cell infiltration was increased in NanoDoce^®^-treated animals compared to untreated, vehicle-treated and IV docetaxel-treated controls. Second, the difference in mature B cells between the bladder and renal models may explain, at least in part, why LS were observed in the tumor-site tissues in the NanoDoce^®^-treated bladder model but not in the NanoDoce^®^-treated renal model. Alternatively, the difference between rat and mouse anatomy may also be responsible for this observation, wherein native LS may be more evident in mice due to a smaller flank area for assessment in comparison to the larger rat. Third, these findings are consistent with other studies of taxanes prepared as submicron particles (paclitaxel: NanoPac^®^) administered by inhalation in a rat orthotopic NSCLC model [4], Verco et al., 2019 in press] and directly into MDA-MB-231 breast cancer xenografts in mice. Finally, the increased immune-cell infiltration coupled with the substantial tumor response may indicate that IT NanoDoce^®^ induces a cytolytic secondary immune response.

Antibody-dependent cell-mediated cytotoxicity (ADCC) and antibody-dependent cell-mediated phagocytosis (ADCP) are processes in the immune response to cancer. DCs and macrophages were presumably present in all xenograft models and were thus available to participate as effector cells in these processes [20]. IV chemotherapy-induced apoptosis often fails to elicit a robust local immune response [21,22]. However, death of cancer cells can be accompanied by changes in the composition of the cell surface and increased availability of soluble tumor-specific antigens [23]. Recent success of IV immunotherapy in a variety of oncology trials [24] suggests that therapy targeting both malignant cells and the tumor-infiltrating immune cells could be effective in eliciting innate and long-lasting adaptive immunity [25,26,27], and disease regression.

## 4. Materials and Methods

### 4.1. Tumor Implantation and Treatment

Animal procedures were conducted under protocols approved by the Institutional Animal Care and Use Committees (IACUC) at University of Kentucky (Lexington, KY, USA) for Hera BioLabs under Project Code UKY 2017-2272 approved in February 2018, at Celsion Labs (Huntsville, AL, USA) under Project Code IACUC-007 approved in October 2017, and Charles River (Morrisville, NC, USA) under Project Code PC3-e19 approved in April 2016.

Clear cell renal carcinomas (786-O human renal cell carcinoma cells (ATCC^®^ CRL-1932™)) were evaluated in 15 female 6–8-week-old Sprague-Dawley Rag2/Il2rg null (SRG™) rats (Hera BioLabs). 5 × 10^6^ cells in 500 µL (5 mg/mL Cultrex BME in DMEM) were delivered subcutaneously to the left hind flank. This study represents the first use of this novel rat model, as such, animal numbers were restricted to three per group by limitations of the breeding colony. Necropsy occurred on Day 57, 50 days post treatment-initiation, unless early euthanasia due to tumor length ≥ 40 mm, tumor weight > 10% of body weight, body weight loss > 20%, or significant tumor ulceration covering > 50% of tumor surface was reached.

Transitional cell bladder carcinomas (UM-UC-3 human urothelial carcinoma cells (ATCC- CRL-1749™)) were evaluated in 47 6–7-week-old female Hsd:Athymic Nude-Foxn1nu mice (Envigo, Madison, WI, USA). 1 × 10^7^ cells in 100 µL (PBS 1:1 with Matrigel) were delivered subcutaneously into the right flank. Necropsy occurred on Day 60, 42 days post treatment-initiation, unless early euthanasia due to tumor volume ≥ 3000 mm^3^ or significant tumor ulceration was reached.

Prostate carcinomas (PC-3 human prostate carcinomas) were evaluated in 60 female 12-week-old Crl:NU(NCr)-Foxn1nu mice (Charles River). A 1 mm^3^ PC-3 (ATCC^®^ CRL-1435™) fragment was implanted subcutaneously on the right flank. Necropsy occurred on Day 86, 60 days post treatment-initiation, unless early euthanasia due to tumor volume ≥ 2000 mm^3^ or weight loss exceeding 30% for one measurement or 25% for three measurements was reached.

Table 1 describes dosing and schedules: IT vehicle (0.2–1% Polysorbate 80/1.6–10% EtOH in 0.9% Sodium Chloride for Injection), IT NanoDoce^®^ and IV docetaxel (administered via the tail vein) were tested in all three tumor types. For the UM-UC-3 and PC-3 mouse studies, the IV dose was 30 mg/kg and in the 786-O rat study 2.5–5.0 mg/kg doses were delivered. IT NanoDoce^®^ dose was 100 mg/kg in the UM-UC-3 xenografts, 37.5 mg/kg or 100 mg/kg in the PC-3 study and 20 mg/kg in the 786-O study.

Technique for IT administration of vehicle and NanoDoce^®^ was designed to maximize distribution of test article throughout the tumor via multiple injection sites. Treatment initiation occurred following randomization, seven days after implant in the 786-O study (mean tumor volume = ~336–427 mm^3^; *n* = 3/group), 18 days after tumor implant in the UM-UC-3 study (mean tumor volume = ~161–164 mm^3^; *n* = 9–10/group) and 26 days after tumor implant (mean tumor volume = ~136–141 mm^3^; *n* = 10/group) in the PC-3 study.

### 4.2. Tumor Volume Evaluations and Analysis

Largest tumor diameter or length (*L*), width (*W*) and height (*H*; UM-UC-3 study only) were measured in millimeters (mm) using manual read or digital calipers 2–4 times weekly through study end. Tumor volume (*V*) was calculated as follows:

$$\begin{array}{l} \mathrm{Renal}\;\mathrm{and}\;\mathrm{prostate}:V\left({\mathrm{mm}}^{3}\right)=\frac{\left(L\times W^{2}\right)}{2}\\ \mathrm{Bladder}:V\left({\mathrm{mm}}^{3}\right)=\frac{4\pi \left(\frac{L}{2}\times \frac{W}{2}\times \frac{H}{2}\right)}{3} \end{array}$$

### 4.3. Statistical Analysis

#### 4.3.1. Tumor Volume

Statistical analysis was performed using studylog^®^ (Studylog Systems, Inc., South San Francisco, CA, USA, 786-O study), or GraphPad Prism 6.07 (GraphPad Software, San Diego, CA, USA), UM-UC-3 and PC-3 study). In the PC-3 study tumor weight was estimated with the assumption that 1 mm^3^ of V is equivalent to 1 mg. In the UM-UC-3 study differences in group mean TVs vs. IT Vehicle or IV docetaxel and in the PC-3 study differences vs. IT Vehicle were compared using one-way ANOVA with Dunnett’s post-test.

#### 4.3.2. Body Weight

Group mean body weights and standard deviation were calculated using Microsoft^®^ Excel (Microsoft Corporation, Redmond, WA, USA). In the 786-O study body weight data is plotted for each group until two or more animals from the group died or were euthanized. In the UM-UC-3 study body weight data is plotted for each group until the first animal from the group died or was euthanized. In the PC-3 study body weight data is plotted for each group until more than two animals from the group died or were euthanized.

### 4.4. Docetaxel Concentration in Tumor Tissues

On the last day the study, if sufficient tissue was available, half of the 786-O and UM-UC-3 tumors were isolated and shipped to Frontage Laboratories Inc. (Exton, PA, USA) for docetaxel content analysis by liquid chromatography-mass spectroscopy (LC-MS/MS). The 786-O tumors were dissected and flash frozen in 2-methylbutane (isopentane), chilled on dry ice and stored at −80 °C. UM-UC-3 tumors were flash frozen in liquid nitrogen and stored at −80 °C.

Docetaxel concentrations were determined using the deuterated analogue, docetaxel-d_9_, as the internal standard. Separation was performed with a Shimadzu LC pump and autosampler (Shimadzu Scientific Instruments, Columbia, MD, USA) using an ACE^®^ C8, 5 μm, 2.1 × 50 mm column (Advanced Chromatography Technologies Ltd., Aberdeen, Scotland) at ambient temperature. An AB/MDS Sciex API 5000 (Applied Biosystems, Foster City, CA, USA) triple quadrupole mass spectrometer equipped with an electrospray ionization source detector was used. The instrument was operated in positive ion mode using multiple reaction monitoring with specific precursor-product ion pairs for docetaxel and docetaxel-d_9_. Concentrations of docetaxel were obtained from calibration curves constructed by plotting the peak area ratios (analyte to internal standard) versus analyte concentration using linear (786-O study) or quadratic (UM-UC-3 study) regression with a weighting of 1/x^2^. A calibration curve, prepared in control tumor tissue homogenate, was analyzed at the beginning and the end of each analytical run. Two sets of quality control (QC) samples were prepared at four concentration levels for the 786-O and three levels for the UM-UC-3 xenografts to ensure reliability of the assay. Standard and QC sample concentrations were deemed acceptable if the calculated concentrations were within ±20% of the nominal concentrations (1.00–2000 ng/g (786-O) and 1.00–1000 ng/g (UM-UC-3); LOQ = 1.00 ng/g).

### 4.5. H&E and IHC Evaluations

The other half of tumor site tissues were assessed by histopathology. UM-UC-3 and 786-O tumor tissues were fixed in 10% neutral buffered formalin for 48–72 h, transferred to 70% ethanol and stored at room temperature. Tissues were paraffin embedded (IDEXX Laboratories, Sacramento, CA, USA) and serially cut. Standard Hematoxylin & Eosin (H&E) staining was performed.

IHC staining was performed on formalin-fixed paraffin-embedded sections by IDEXX BioAnalytics (Columbia, MO, USA). All procedures, except for heated tissue pretreatments, were performed at room temperature. The CD68 marker was optimized for both the 786-O (rat) and UM-UC-3 (mouse) xenografts. CD11b and the pan-cytokeratin marker AE1/AE3 were successfully optimized for the 786-O (rat) but not the UM-UC-3 (mouse) xenografts. BCL-6 staining was only performed on UM-UC-3 tissue samples. An unblinded, board-certified pathologist reviewed all slides. In all cases, following primary antibody incubation, sections were rinsed and incubated with detection system for 30 min, washed again, then incubated with 3,3′-Diaminobenzidine (DAB) or NovaRed substrate for 5–10 min.

CD11b detection used the Envision system (Agilent/DAKO; Carpinteria, CA, USA) with control rat spleen tissue. Sections were pre-treated with heated 10 mM citrate buffer (pH 6.0), rinsed and incubated for 5 min. with wash buffer (Tris Saline buffer with Tween 20, pH 7.6). Following a 5-min. treatment with 3% H_2_O_2_, sections were rinsed with wash buffer and then FC-receptor block was applied for 30 min. Sections were incubated with primary antibody anti-CD11b (polyclonal, produced in rabbits; product #110-89747; Novus Biologicals USA; Littleton, CA, USA) for 60 min. at 1:1200 dilution.

Pan-cytokeratin detection used the biotinylated goat anti-mouse IgG with streptavidin:HRP (Jackson Immunolabs; West Grove, PA, USA) with control rat skin. Sections were pre-treated with heated 10 mM citrate buffer (pH 6.0) and rinsed and incubated for 5 min. with wash buffer. Following a 5 min. treatment with 3% H_2_O_2_, sections were rinsed with wash buffer and then blocked with 5% BSA for 20 min. Sections were incubated with pan-cytokeratin (clone AE1:AE3, produced in mice; product #M3515; Agilent/ DAKO) for 60 min. at 1:50 dilution.

CD68 detection in the 786-O xenografts used the biotinylated goat anti-mouse IgG with streptavidin:HRP (Jackson Immunolabs) with control rat spleen tissue. Sections were pre-treated with proteinase K (diluted) and then rinsed and incubated for 5 min. with wash buffer. Following a 5 min. treatment with 3% H_2_O_2_, sections were rinsed with wash buffer and then blocked with 5% BSA for 20 min. Sections were incubated with anti-CD68 (clone ED1, produced in mice; product #MCA341R; AbD Serotec; Raleigh, NC, USA) antibodies for 60 min. at 1:200 dilution.

CD68 detection in the UM-UC-3 xenografts used the biotinylated rabbit anti-rat IgG with streptavidin:HRP (Jackson Immunolabs) with control mouse spleen tissue. Sections were pre-treated with 10 mM citrate buffer (pH 6.0) and then rinsed and incubated for 5 min. with wash buffer. Following a 5 min. treatment with 3% H_2_O_2_, sections were rinsed with wash buffer and then blocked with 5% BSA for 20 min. Sections were incubated with anti-CD68 (clone FA-11, produced in rats; product #MCA 1957; AbD Serotec; Raleigh, NC, USA) antibodies for 60 min. at 1:200 dilution.

BCL-6 detection in the UM-UC-3 xenografts used the Envision system (Agilent/DAKO) with control mouse spleen lymphoma tissue. Sections were pre-treated with 10 mM citrate buffer (pH 6.0) and rinsed and incubated for 5 min. with wash buffer. Following a 5 min. treatment with 3% H_2_O_2_, sections were rinsed with wash buffer and then blocked with 5% BSA for 20 min. Sections were incubated with anti-BCL-6 (polyclonal, produced in rabbit; product #NBP2-59786; Novus Biologicals USA; Centennial, CO, USA) antibodies for 60 min. at 1:100 dilution followed by DAB substrate incubation and Mayer’s hematoxylin staining.

## 5. Conclusions

The prolonged residence of submicron particle docetaxel within the tumor microenvironment may facilitate tumor cell death, concentrating tumor antigens while eliminating immuno-suppressive tumor cells. This theory is supported by our observations of eradication or reduction of tumor in IT NanoDoce^®^-treated xenografts within the nearly 2-month study durations. The accompanying high levels of docetaxel in tumor-site tissues confirm the extended residence time. In contrast, tumor growth briskly recurred in the bladder cancer xenograft after cessation of IV docetaxel treatment and little to no docetaxel was detected in tumor tissues. The immune-cell response, as detected by the increase in CD68^+^ and CD11b^+^ cells in tumor-site tissues from the various xenografts, supports the hypothesis that IT NanoDoce^®^ administration initiates a chain of events involving both tumor cell death via direct cytotoxic effects and indirect stimulation of effector immune cells. Clinical trials are underway to evaluate local NanoDoce^®^ administration by cystoscopic injection into bladder tumor sites and are planned for direct injection of renal tumors.

## Figures and Tables

**Table 1:** Genitourinary-oncologic Xenograft Study Treatment Schedules.

Study	*n*	Treatment	Dose	Route	Schedule	NanoDoce^®^ Designation
Renal Cancer Xenograft (786-O in rat)	3	Untreated	N/A	N/A	N/A	-
	3	Vehicle	-	IT	qweekly 3×	-
	3	Docetaxel	2.5–5 mg/kg	IV	qweekly 2–3×	-
	3	NanoDoce^®^	20 mg/kg	IT	qweekly 1×	1×
	3		20 mg/kg	IT	qweekly 2×	2×
	3		20 mg/kg	IT	qweekly 3×	3×
Bladder Cancer Xenograft (UM-UC-3 in mouse)	1	Untreated	N/A	N/A	N/A	-
	10	Vehicle	-	IT	qweekly 3×	-
	9	Docetaxel	30 mg/kg	IV	qweekly 3×	-
	10	NanoDoce^®^	100 mg/kg	IT	qweekly 1×	1×
	9		100 mg/kg	IT	qweekly 2×	2×
	9		100 mg/kg	IT	qweekly 3×	3×
Prostate Cancer Xenograft (PC-3 in mouse)	10	Vehicle	-	IT	qweekly 3×	-
	10	Docetaxel	30 mg/kg	IV	qweekly 3×	-
	10	NanoDoce^®^	100 mg/kg	IT	qweekly 1×	Low
	10		37.5 mg/kg	IT	qweekly 3×	Medium
	10		100 mg/kg	IT	qweekly 3×	High

## Supplementary Materials

The following are available online at https://www.mdpi.com/2072-6694/11/4/577/s1, Table S1: Degree of tumor necrosis in renal cancer cases, Table S2: Degree of tumor necrosis in bladder cancer cases, Table S3: Inflammatory cell infiltrate density per treatment group in renal cancer cohort, Table S4: Macrophage infiltrate density in bladder cancer per treatment group.

## References

[1] Goldberg E.P., Hadba A.R., Almond B.A., Marotta J.S. (2002). Intratumoral cancer chemotherapy and immunotherapy: Opportunities for nonsystemic preoperative drug delivery. J. Pharm. Pharmacol..

[2] Zhao M., Liu M. (2018). New Avenues for Nanoparticle-Related Therapies. Nanoscale Res. Lett..

[3] Williamson S.K., Johnson G.A., Maulhardt H.A., Moore K.M., McMeekin D.S., Schulz T.K., Reed G.A., Roby K.F., Mackay C.B., Smith H.J. (2015). A phase I study of intraperitoneal nanoparticulate paclitaxel (Nanotax(R)) in patients with peritoneal malignancies. Cancer Chemother. Pharmacol..

[4] Verco J., Johnston W., Baltezor M., Kuehl P.J., Gigliotti A., Belinsky S.A., Lopez A., Wolff R., Hylle L., diZerega G. (2018). Pharmacokinetic Profile of Inhaled Submicron Particle Paclitaxel (NanoPac((R))) in a Rodent Model. J. Aerosol Med. Pulm. Drug Deliv..

[5] Zitvogel L., Kepp O., Kroemer G. (2010). Decoding cell death signals in inflammation and immunity. Cell.

[6] Zitvogel L., Kepp O., Kroemer G. (2011). Immune parameters affecting the efficacy of chemotherapeutic regimens. Nat. Rev. Clin. Oncol..

[7] Galluzzi L., Senovilla L., Zitvogel L., Kroemer G. (2012). The secret ally: Immunostimulation by anticancer drugs. Nat. Rev. Drug Discov..

[8] Liu W.M., Fowler D.W., Smith P., Dalgleish A.G. (2010). Pre-treatment with chemotherapy can enhance the antigenicity and immunogenicity of tumours by promoting adaptive immune responses. Br. J. Cancer.

[9] Kroemer G., Galluzzi L., Kepp O., Zitvogel L. (2013). Immunogenic cell death in cancer therapy. Annu. Rev. Immunol..

[10] Krysko D.V., Vandenabeele P. (2010). Clearance of dead cells: Mechanisms, immune responses and implication in the development of diseases. Apoptosis.

[11] Ravichandran K.S. (2011). Beginnings of a good apoptotic meal: The find-me and eat-me signaling pathways. Immunity.

[12] Locher C., Conforti R., Aymeric L., Ma Y., Yamazaki T., Rusakiewicz S., Tesniere A., Ghiringhelli F., Apetoh L., Morel Y. (2010). Desirable cell death during anticancer chemotherapy. Ann. N. Y. Acad. Sci..

[13] Bracci L., Schiavoni G., Sistigu A., Belardelli F. (2014). Immune-based mechanisms of cytotoxic chemotherapy: Implications for the design of novel and rationale-based combined treatments against cancer. Cell Death Differ..

[14] Millrud C.R., Mehmeti M., Leandersson K. (2018). Docetaxel promotes the generation of anti-tumorigenic human macrophages. Exp. Cell Res..

[15] Hodge J.W., Garnett C.T., Farsaci B., Palena C., Tsang K.Y., Ferrone S., Gameiro S.R. (2013). Chemotherapy-induced immunogenic modulation of tumor cells enhances killing by cytotoxic T lymphocytes and is distinct from immunogenic cell death. Int. J. Cancer.

[16] Chen J., Yuan L., Fan Q., Su F., Chen Y., Hu S. (2012). Adjuvant effect of docetaxel on the immune responses to influenza A H1N1 vaccine in mice. BMC Immunol..

[17] Kodumudi K.N., Woan K., Gilvary D.L., Sahakian E., Wei S., Djeu J.Y. (2010). A novel chemoimmunomodulating property of docetaxel: Suppression of myeloid-derived suppressor cells in tumor bearers. Clin. Cancer Res..

[18] Tsavaris N., Kosmas C., Vadiaka M., Kanelopoulos P., Boulamatsis D. (2002). Immune changes in patients with advanced breast cancer undergoing chemotherapy with taxanes. Br. J. Cancer.

[19] Engelhard V.H., Rodriguez A.B., Mauldin I.S., Woods A.N., Peske J.D., Slingluff C.L. (2018). Immune Cell Infiltration and Tertiary Lymphoid Structures as Determinants of Antitumor Immunity. J. Immunol..

[20] Roman V.R.G., Murray J.C., Weiner L.M., Ackerman M.E., Nimmerjahn F. (2014). Antibody-Dependent Cellular Cytotoxicity (ADCC). Antibody Fc: Linking Adaptive and Innate Immunity.

[21] Zitvogel L., Kepp O., Senovilla L., Menger L., Chaput N., Kroemer G. (2010). Immunogenic tumor cell death for optimal anticancer therapy: The calreticulin exposure pathway. Clin. Cancer Res..

[22] Green D.R., Ferguson T., Zitvogel L., Kroemer G. (2009). Immunogenic and tolerogenic cell death. Nat. Rev. Immunol..

[23] Zitvogel L., Galluzzi L., Smyth M.J., Kroemer G. (2013). Mechanism of action of conventional and targeted anticancer therapies: Reinstating immunosurveillance. Immunity.

[24] Brahmer J.R., Tykodi S.S., Chow L.Q., Hwu W.J., Topalian S.L., Hwu P., Drake C.G., Camacho L.H., Kauh J., Odunsi K. (2012). Safety and activity of anti-PD-L1 antibody in patients with advanced cancer. New Engl. J. Med..

[25] Marciscano A.E., Madan R.A. (2018). Targeting the Tumor Microenvironment with Immunotherapy for Genitourinary Malignancies. Curr. Treat. Options Oncol..

[26] Schumacher T.N., Schreiber R.D. (2015). Neoantigens in cancer immunotherapy. Science.

[27] Nelson D., Fisher S., Robinson B. (2014). The “Trojan Horse” approach to tumor immunotherapy: Targeting the tumor microenvironment. J. Immunol. Res..

