# Supplementary material for: Local Injection of Submicron Particle Docetaxel is Associated with Tumor Eradication, Reduced Systemic Toxicity and an Immunologic Response in Uro-Oncologic Xenografts
Source: Cancers (Basel). 2019 Apr 24;11(4):577. doi: 10.3390/cancers11040577 (PMC6520999; doi:10.3390/cancers11040577)
Supplement: Supplementary file 1 [file cancers-11-00577-s001.pdf]

Supplementary Material

Local Injection of Submicron Particle Docetaxel is Associated with Tumor Eradication, Reduced Systemic Toxicity and an Immunologic Response in Uro-oncologic Xenografts

Holly Maulhardt, Lauren Hylle, Michael Frost, Ashley Tornio, Sara Dafoe, Leanne Drummond, David I. Quinn, Ashish Kamat and Gere diZerega

Table S1. Inflammatory cell infiltrate density per treatment group in renal cancer study.

Treatment		Total # of Animals	Mild	Moderate	Marked
Non-NanoDoce®-Treated	Non-treated Control	2	2	-	-
	IT vehicle 3×	2	2	-	-
	IV Docetaxel 3×	2	2	-	-
NanoDoce®-Treated	IT NanoDoce® 1×	3	2	1	-
	IT NanoDoce® 2×	3	-	3	-
	IT NanoDoce® 3×	2	-	1	1

Semi-quantitative H&E and IHC (anti-CD11b) assessment of lymphohistiocytic infiltrate density. Among non-NanoDoce®-treated animals, a mild lymphohistiocytic infiltrate was present in the peritumoral non-neoplastic stroma without overt inflammation within the tumor. By contrast, five of the eight animals in the IT NanoDoce® groups contained a moderate lymphohistiocytic infiltrate and one had a marked lymphohistiocytic infiltrate. This correlated with the increased amount of necrosis in the IT NanoDoce®-treated animals.

Table S2. Degree of tumor necrosis in renal cancer study.

Treatment		Total # of Animals	100%	>90%	50–90%	5–50%	< 5%
Non-NanoDoce®-Treated	Non-treated Control	2	-	-	-	-	2
	IT vehicle 3×	2	-	-	-	-	2
	IV Docetaxel 3×	2	-	-	-	-	2
NanoDoce®-Treated	IT NanoDoce® 1×	3	1	-	-	-	2 *
	IT NanoDoce® 2×	3	1	1	1	-	-
	IT NanoDoce® 3×	2	2	-	-	-	-

* Surrounding tissue not available in sufficient quantity for definitive necrosis assessment. One of these did contain a focal rim of necrosis that represented <5% of the submitted tissue area. Tumor necrosis in the non-NanoDoce®-treated groups consisted of small discrete foci of necrosis in the tumor that occupied < 5% of the tumor area, and located within central portions of the tumor nodule, suggesting these may be secondary to hypoxemia.

Table S3. Degree of tumor necrosis in bladder cancer study.

Treatment		Total # of Animals	100%	>90%	50–90%	11–50%	5–10%	< 5%
Non-NanoDoce®-Treated	Non-treated Control	1	-	-	-	-	1	-
	IT vehicle 3×	2	-	-	1	1	-	-
	IV Docetaxel 3×	2	-	-	1	1	-	-
NanoDoce®-Treated	IT NanoDoce® 1×	3	-	-	1	2	-	-
	IT NanoDoce® 2×	5	2	2	-	-	1	-
	IT NanoDoce® 3×	3	3	-	-	-	-	-

Tumor cell necrosis in the non-NanoDoce-treated animals included two with 50–90% tumor necrosis. Overall, the extent of tumor cell necrosis was greater in the NanoDoce-treated groups than in the non-NanoDoce-treated groups.

Table S4. Macrophage infiltrate density in bladder cancer study.

Treatment		Total # of Animals	Mild	Moderate	Marked
Non-NanoDoce®-Treated	Non-treated Control	1	1	-	-
	IT vehicle 3×	2	2	-	-
	IV Docetaxel 3×	2	2	-	-
NanoDoce®-Treated	IT NanoDoce® 1×	3*	2	-	-
	IT NanoDoce® 2×	5	4	1	-
	IT NanoDoce® 3×	3	-	3	-

* One of these had no surrounding non-neoplastic tissue available for assessment. Semi-quantitative H&E and IHC (anti-CD68) assessment of macrophage infiltrate density in surrounding non-neoplastic tissue. The intensity of the macrophage infiltrate in the surrounding non-neoplastic tissue was not striking in any of the animals; however, when the non-NanoDoce®-treated groups were compared to the NanoDoce®-treated groups, it was noted that the latter contained cases with a moderate degree of macrophage infiltrate while this was not seen in the non-NanoDoce®-treated groups.

© 2019 by the authors. Licensee MDPI, Basel, Switzerland. This article is an open access article distributed under the terms and conditions of the Creative Commons Attribution (CC BY) license (http://creativecommons.org/licenses/by/4.0/).
